# Clinical features of excyclotorsion in the non-paretic eye of patients with congenital unilateral superior oblique palsy

**DOI:** 10.1186/s12886-022-02339-5

**Published:** 2022-03-16

**Authors:** Eun-Hyang Cha, Suk-gyu Ha, Youngwoo Suh Shu, Seung-Hyun Kim

**Affiliations:** Department of Ophthalmology, College of Medicine, Korea University Guro Hospital, 148, Gurodong-ro, Guro-gu, Seoul, 08308 Republic of Korea

**Keywords:** Age, Excyclotorsion, Fixation, Surgery, Superior oblique palsy

## Abstract

**Background:**

To investigate preoperative clinical features and postoperative results according to the correspondence between excyclotorsion and the paretic eye in patients with congenital unilateral superior oblique palsy (USOP).

**Methods:**

A retrospective review of medical charts was performed. The patients were divided into the accordance (ocular excyclotorsion in the paretic eye) and disaccordance (ocular excyclotorsion in the non-paretic eye) groups. The degree of excyclotorsion (scale, 0–4) was measured. Age, sex, hypertropia at the primary position, fixation preference, inferior oblique overaction, and degree of excyclotorsion were measured.

**Results:**

Ninety-eight patients were included in this study. There were 70 (71.4%) and 28 patients (28.6%) in the accordance and disaccordance groups. Sixteen patients (22.9%) in the accordance group and 12 patients (42.9%) in the disaccordance group were aged under 2 years (*p* = 0.04). A fixation preference of the paretic eye was observed in 2 (2.9%) and 8 (28.6%) patients in the accordance and disaccordance groups (p < 0.01). The postoperative degree of excyclotorsion in the accordance group (0.14 ± 0.39) was lower than that in the disaccordance group (0.28 ± 0.71) (*p* = 0.01). The residual postoperative excyclotorsion (> 1) were observed in the disaccordance group (14 patients, 50%) and accordance group (16 patients, 22.9%) (*p* = 0.01).

**Conclusion:**

Preoperative disaccordance between excyclotorsion and the paretic eye was observed in patients who were under 2 years of age and preferred fixation of the paretic eye. The postoperative degree of excyclotorsion was lower in the accordance group.

## Introduction

Unilateral superior oblique palsy (USOP) may be congenital or acquired. Congenital USOP is the most common form of USOP, accounting for approximately 33–69.2% of the cases. A common cause of acquired USOP is trauma followed by vascular diseases, such as hypertension, diabetes, tumor, iatrogenic factors, and congenital conditions [1].

Congenital USOP is one of the most common forms of paralytic cyclovertical strabismus [2–5], which can be accompanied by under- depression of the superior oblique and overaction of the ipsilateral inferior oblique muscle. Abnormal head posture and facial asymmetry could be strong evidence of USOP in patients presenting with a positive Bielschowsky head-tilt test result [6–8]. The degree of facial asymmetry is usually proportional to the degree of head-tilt such that early strabismus surgery to correct the head-tilt may help prevent facial asymmetry in congenital SOP. Patients with USOP typically have hypertropia of the paretic eye, which is combined with horizontal strabismus in 72.2% of the cases [9].

As the primary function of the superior oblique is incyclotorsion, paralysis of the muscle can cause significant ocular excyclotorsion of the paretic eye, which is one of the typical clinical manifestations of USOP. However, excyclotorsion is not always present in the paretic eye. Several studies have found that approximately 25% of the patients with USOP had ocular excyclotorsion in the non-paretic eye [10–13].

Although it is unclear why ocular excyclotorsion is present in non-paretic eyes, several studies have found that fixation preference [14] and neural adaptation [15–17] may have a clinically significant influence on ocular excyclotorsion in non-paretic eyes.

In this study, we aimed to assess the clinical features associated with the correspondence of paretic eyes and excyclotorsion in USOP, and investigate postoperative changes in ocular excyclotorsion in USOP.

## Material and methods

### Patients

This study was approved by the Institutional Review Board of Korea University Medical Center. It adhered to tenets of the Declaration of Helsinki. Written informed consent was obtained from all patients and their guardians. We retrospectively reviewed the medical records of 98 consecutive patients with congenital USOP diagnosed between 2001 and 2015 at the Department of Ophthalmology, Korea University Medical Center. The diagnosis of USOP was based on hypertropia, apparent elevation and under-depression in adduction of the paretic eye, anomalous head posture, or a positive Bielschowsky head-tilt test. All enrolled patients underwent full ophthalmic examination including visual acuity, refraction test, ocular movement, fixation preference [18], scale of superior oblique under- depression (SOUA) and inferior oblique overaction(IOOA), angle of deviation in the alternate prism cover test (prism diopters, PD), the Bielschowsky head-tilt test, presence of amblyopia or dissociated vertical deviation (DVD), and fundus examination.

Amblyopia was defined as a best-corrected visual acuity of 6/9 or worse that was not directly attributable to any underlying structural abnormality of the eye or visual pathway. The exclusion criteria included acquired (masked) bilateral superior oblique palsy during ophthalmic examination, previous ocular surgery, history of head trauma, cerebrovascular diseases, and neurological disorders.

The SOUA and IOOA were assessed using a 0 to 4 scale. The scales were documented before the operation and at each postoperative visit. The scale were graded the difference between the corneal limbus height in each eye according to the degree of over-elevation of the eye in adduction for IOOA and of under- depression of the eye in adduction for SOUA [19]. The over-elevation and the under- depression scale ranged from 0 to 4, respectively.

Fundus examination was performed to measure the degree of excyclotorsion using a fundus camera (TRC-50DX, Topcon Medical System, Tokyo, Japan) or indirect ophthalmoscope before and after the operation. The degree of excyclotorsion was measured using the Guyton grading scale [20]. Values + 1 to + 4 represent excyclotorsion and values − 1 to − 4 represent incyclotorsion based on the classification. When a horizontal line passed through the fovea within the inferior one-third of the disc, it was considered a normal range of torsion. If the vertical position of the fovea was located below the center of the optic disc, the excyclotorsion was graded from + 1 to + 4.

Surgical procedures performed in this study included inferior oblique recession (12–14 mm) with or without superior rectus recession (> 15 PD of hypertropia in the primary position) based on the preoperative amount of hypertropia at the primary position. Postoperative examinations were performed at 1, 3, 6, and 12 months, and then routinely at 1-year intervals. All measurements were compared between pre- and postoperative final visits. Patients who were followed-up for at least 6 months postoperatively were included.

All patients were divided into the accordance (preoperative fundus ocular excyclotorsion in the paretic eye) and disaccordance (preoperative ocular excyclotorsion in the non-paretic eye) groups. The pre- and postoperative clinical features and the degree and change in excyclotorsion were compared between the accordance and disaccordance groups.

### Statistical analysis

Statistical analysis was conducted using SPSS version 21.0 (IBM Corporation, Armonk, NY, USA). To assess the differences between the two groups, we used the Mann–Whitney test and Fisher’s exact test. Statistical significance was set at p < 0.05.

## Results

A total of 98 patients who met the eligibility criteria were included in the study. There were 70 patients (71.4%) in the accordance group and 28 patients (28.6%) in the disaccordance group. The mean ages in the accordance and disaccordance groups were 9.2 ± 12.7 years (1–62 years) and 7.5 ± 8.9 years (1–31 years), respectively (*p* = 0.17). Hypertropia in the primary position was 13.1 ± 12.8 PD (5–35 PD) in the accordance group and 13.0 ± 9.1 PD (4–45 PD) in the disaccordance group (*p* = 0.82) (Table 1). There was no statistically significant difference between the two groups with regard to sex and the prevalence of amblyopia and DVD (p > 0.05, all). There were 16 (22.9%) and 12 (42.9%) patients aged < 2 years in the accordance and disaccordance groups, respectively (*p* = 0.04). Table 1 shows the demographic details of the participants.

**Table 1:** Basic demographics

	Accordance group	Disaccordance group	*p*
Number of patients, n (%)	70 (71.4)	28 (28.6)	< 0.01^a^
Age, years, n (%)	9.2 ± 12.7 (1–62)	7.5 ± 8.9 (1–31)	0.17^a^
< 2	16 (22.9)	12 (42.9)	0.04^b^
2–10	26 (37.1)	6 (21.4)	
11–17	20 (28.6)	4 (14.3)	
≥ 18	8 (11.4)	6 (21.4)	
Male sex, n (%)	53 (75.7)	17 (60.7)	0.11^b^
Amblyopia, n (%)	3 (4.5)	nil	0.35^b^
DVD, n (%)	2 (2.8)	2 (7.1)	0.29^b^

^a^Mann–Whitney test

^b^Fisher’s exact test, *DVD* Dissociated vertical deviation

Two patients (2.9%) and eight patients (28.6%) in the accordance and disaccordance groups, respectively, had a fixation preference in the paretic eye (*p* < 0.01). The preoperative degree of excyclotorsion (2.3 ± 0.67, 1–4) in the accordance group was significantly greater than that in the disaccordance group (1.9 ± 0.64, 1–4) (*p* = 0.03). Table 2 shows the clinical features in two groups.

**Table 2:** Clinical characteristics in the accordance and discordance groups

Parameters	Accordance group	Disaccordance group	*p*
Fixation preference on paretic eye	2 (2.9)	8 (28.6)	< 0.01^b^
Horizontal deviation	15 (21.4)	8 (28.6)	0.31^b^
Hypertropia at primary position, PD	13.1 ± 12.8 (5–35)	13.0 ± 9.1 (4–45)	0.82^a^
Scale of SOUA	1.7 ± 0.8 (1–3.5)	1.6 ± 0.9 (1–3)	0.76^a^
Scale of IOOA	2.3 ± 0.8 (0.5–3.5)	2.5 ± 0.8 (1–4)	0.23^a^
Degree of excyclotorsion	2.3 ± 0.67 (1–4)	1.9 ± 0.64 (1–4)	0.03^a^

^a^Mann–Whitney test

^b^Fisher’s exact test

*PD* Prism diopters, *SOUA* Superior oblique underaction, *IOOA* Inferior oblique overaction

The postoperative excyclotorsion was 0.14 ± 0.39 (0–3) in the accordance group and 0.28 ± 0.71 (0–3) in the disaccordance group (*p* = 0.18). Fifty patients (71.4%) in the accordance group and 11 patients (39.3%) in the disaccordance group had a postoperative reduction in excyclotorsion > 1 (*p* = 0.01) (Table 3).

**Table 3:** Postoperative results in the accordance and discordance groups

	Accordance group	Disaccordance group	*p*
Hypertropia in the primary position, PD	1.31 ± 3.32 (0–10)	1.06 ± 2.61 (0–16)	0.52^a^
Scale of SOUA	0.23 ± 0.52 (0–3.5)	0.12 ± 0.23 (0–1.5)	0.11^a^
Scale of IOOA	0.15 ± 0.37 (0–2)	0.21 ± 0.52 (0–1)	0.51^a^
Degree of excyclotorsion	0.14 ± 0.39 (0–3)	0.28 ± 0.71 (0–3)	0.18^a^
Excyclotorsion reduction (> 1) n, (%)	50 (71.4)	11 (39.3)	0.01^b^
Residual (> 1) excyclotorsion n, (%)	16 (22.9)	14 (50)	0.01^b^

^a^Mann–Whitney test

^b^Fisher’s exact test

*PD* Prism diopters, *SOUA* Superior oblique underaction, *IOOA* Inferior oblique overaction

There were 14 patients (50%) with postoperative residual excyclotorsion (> 1) in the disaccordance group, compared with 16 patients (22.9%) in the accordance group (*p* = 0.01). Table 3 shows the postoperative results in the accordance and disaccordance groups.

## Discussion

Patients with USOP generally demonstrate hypertropia and excyclotorsion in the paretic eye. In this study, the excyclotorsion of USOP was more frequently observed in the paretic eye (71.4%) than in the non-paretic eye (28.6%). This is consistent with previous studies showing that 25% of cases of excyclotorsion could also be present in the non-paretic eye [12, 13, 21].

In this study, the incidence of paradoxical ocular excyclotorsion in the non-paretic eye could be increased when patients had a fixation preference in the paretic eye or were aged < 2 years. Initially, paralysis of the superior oblique may lead to ocular excyclotorsion in the paretic eye, followed by ocular dominance [8], cyclofusion [15], or a neural adaptation mechanism [16] in the non-paretic eye, which can induce ocular excyclotorsion in either eye.

According to Kim et al. [14], the paretic dominant eye could influence the alignment of the non-paretic eye through conjugate cycloversion eye movements. It has been demonstrated that neural adaptability for improving the efficiency of motor control and visual function may contribute to the torsional state in USOP. Prolonged fixation with the paretic dominant eye might act in a way that conversely decreases the amount of extortion in the eye, which induces excyclotorsion in the non-paretic eye through conjugate movements according to Hering’s law [17]. Additionally, Kushner and Hairharan reported that long-term fixation with the affected eye in USOP could induce fundus torsional change in the affected eye [22].

In this study, 42.9% of patients with excyclotorsion in the non-paretic eye were under 2 years of age. This result could be explained by the immature binocular visual system and sensory adaptation of children. A lack of immediate motor correction for torsional misalignment and defective binocular fusion in USOP may disrupt cyclofusion, which, in turn, induces the eye to be more excyclorotated, especially in the case of a non-paretic eye. Graf et al. demonstrated that the resting position of human eyes became more excyclorotated after disrupting binocular fusion with 8 h of consecutive monocular occlusion [23]. Shin et al. also demonstrated that a certain level of defective binocular fusion might disrupt cyclofusion and subsequently make the eyes more excyclorotated [24].

In an effort to overcome vertical diplopia, patients with poor sensory adaptation, especially children, would have a greater need for vertical fusion. The immaturity of fusion would contribute to excycloduction in the non-paretic eye. Repetitive sensorial and motor adaptations to torsional misalignment aggravate fundus extorsion in patients with USOP under 2 years of age.

This study demonstrates that the reduction of excyclotorsion after surgery was larger in the accordance group than in the disaccordance group, consistent with the results of a previous study [10]. A possible explanation for this result might be a significant improvement in excyclotorsion in the paretic eye, which might be due to postoperative mechanical changes in the extraocular muscles, which has a direct effect on the torsional forces in the accordance group. In previous study, combined horizontal as the clinical factor in accordance group was observed [10]. However, prevalence of combined horizontal deviations were no different between two groups in this study. We speculated that several clinical characteristics, such as involving low proportion of patients who were aged under 2 years and patients with acquired SOP, may affect the prevalence of combined horizontal deviations.

This study has a few limitations. First, it was conducted retrospectively, and most patients were pediatric patients. Second, although 98 patients were enrolled in this study, additional USOP patients might be needed to confirm the findings of this study. Third, the possibility of observer bias might exist for measurements of pre- and postoperative degrees of excyclotorsion using fundus examination in patients who were under 2 years of age. Lastly, actual number of patients under 2 years old and incidence of fixation preference on paretic eye were different in 2 groups. Unequal sample size of patients under 2 years old and fixation preference on paretic eye between 2 groups (accordance and disaccordance group) may affect the statistical results in this study. Further study with larger and equal sample size in 2 groups may be required.

In conclusion, the paretic eye may not coincide with excyclotorsion in USOP. Excyclotorsion in the non-paretic eye was observed in 28% of patients with USOP. Patients aged < 2 years and with a fixation preference for the paretic eye might have a significantly greater propensity to have excyclotorsion in the non-paretic eye. Surgery for USOP can have a more obvious effect in improving excyclotorsion in the paretic eye.

## Data Availability

The datasets used and/or analysed during the current study available from the corresponding author on reasonable request.
